# Supplementary material for: Multiple antagonist calcium-dependent mechanisms control CaM kinase-1 subcellular localization in a C. elegans thermal nociceptor
Source: eLife. 2023 May 11;12:e85260. doi: 10.7554/eLife.85260 (PMC10174685; doi:10.7554/eLife.85260)
Supplement: Supplementary file 1. [file elife-85260-supp1.docx]

**Supplementary File 1**

| **Plasmid types** | **Plasmid name, cloning and primer information** |
| --- | --- |
| Promoter plasmids (Multi-Site Gateway slot 1): | The generation of **dg68***[slot1 Entry mec-3p(noATG)]* and **dg229***[slot1 Entry QUASprom]* was previously described in Schild and Glauser, 2015. |
| Plasmids with *cmk-1* point mutations and primers used for site-directed mutagenesis: | The generation of **dg588***[slot2 Entry cmk-1(K71A/R74Q/R77S)],* **dg589***[slot2 Entry cmk-1(W305S)],* **dg590***[slot2 Entry cmk-1(V292A/V294A)],* **dg591***[slot2 Entry cmk-1(L321A/L323A)],* **dg593***[slot2 Entry cmk-1(K307Q)],* **dg658***[slot2 Entry cmk-1(K71A/R74Q/R77S/V292A/V294A)]* was previously described in Ippolito *et al.* 2021. The generation of **dg214** *[slot2 Entry cmk-1(T179A)]* was previously described in Schild *et al.* 2014.  **dg592***[slot2 Entry cmk-1(T179D)]*  T179D_F: gacGCGTGTGGAACACCGGGATA  T179D_R: AGCCATTACTCCTGAATCTTCGGTTTTTG  **dg661***[slot2 Entry cmk-1(K71A/R74Q/R77S/T179D)]*  T179D_F/R with dg588 (Ippolito *et al.* 2021) as template  **dg662***[slot2 Entry cmk-1(W305S/T179D)]*  W305S_F/R (Ippolito *et al.* 2021) with dg592 as template  **dg664***[slot2 Entry cmk-1(T179A/V292A/V294A)]*  NES^288-294^_F/R (Ippolito *et al.* 2021) with dg214 as template  **dg692***[slot2 Entry cmk-1(T179A/W305S)]*  W305S_F/R (Ippolito *et al.* 2021) with dg214 as template  **dg932***[slot2 Entry cmk-1(S325A)]*  S325A_F: gCcAATAGCAATCGCCTACAGAAACAAGCT  S325_R: GGAGAGACGGAGCATTTGAAGCT  **dg933***[slot2 Entry cmk-1(S325D)]*  S325D_F: gacAATAGCAATCGCCTACAGAAACAAGCT  S325_R: GGAGAGACGGAGCATTTGAAGCT  **dg998***[slot2 Entry cmk-1(T179D/V292A/V294A)]*  T179D_F/R with dg590 (Ippolito *et al.* 2021) as template  **dg1000***[slot2 Entry cmk-1(K71A/R74Q/R77S/V292A/V294A/L321A/L323A)]*  NES^314-323^_F/R (Ippolito *et al.* 2021) with dg658 as template  **dg1001***[slot2 Entry cmk-1(K71A/R74Q/R77S/V292A/V294A/K307Q)]*  NLS^297-308^_F/R (Ippolito et al.2021) with dg658 as template |
| Plasmid for ckk-1 expression | **dg350** *[slot2 Entry ckk-1b (with ATG noSTOP)]* was created by BP recombination into pDONR_221 of a PCR product generated with  attB1ckk-1a_F ggggacaagtttgtacaaaaaagcaggcttaatgtacacatttcagtcggtctcaca  attB2ckk-1_R ggggaccactttgtacaagaaagctgggtcctattgtctagcggactcgacttttg  **dg287***[slot2 Entry ckk-1b (with ATG noSTOP)]* was created by BP recombination into pDONR_221 of a PCR product generated with  attB1ckk-1b_F ggggacaagtttgtacaaaaaagcaggcttaatgaaaaacacgttcgcacgatact  attB2ckk-1_R ggggaccactttgtacaagaaagctgggtcctattgtctagcggactcgacttttg |
| 3’ UTR and tagging plasmids (Multi-site Gateway slot 3) | **mg211** *[slot3 Entry unc-54 3’UTR]* (aka pMH473) was a gift from Marc Hammarlund.  **dg397** *[slot3 Entry mNG::3xFLAG::unc-54 3’UTR]* previously described in (Hostettler *et al.* 2017). |
| Selection markers used for transgenesis | **dg9** [coel::RFP] *(or unc-122p::RFP)* was a gift from Piali Sengupta (Addgene plasmid # 8938) |
| Expression plasmids used for transgenesis with a description of their creation | **dg405***[mec-3p::cmk-1::mNG::3xFlag]* was previously described in (Hostettler *et al.* 2017).  **dg605***[mec-3p::cmk-1(K71A/R74Q/R77S)::mNG::3xFlag::unc-54 3’UTR]* was created through a LR recombination reaction between dg68, dg588, dg397 and pDEST-R4-P3.  **dg606***[mec-3p::cmk-1(W305S)::mNG::3xFlag::unc-54 3’UTR]* was created through a LR recombination reaction between dg68, dg589, dg397 and pDEST-R4-P3.  **dg607***[mec-3p::cmk-1(V292A/V294A)::mNG::3xFlag::unc-54 3’UTR]* was created through a LR recombination reaction between dg68, dg590, dg397 and pDEST-R4-P3.  **dg665***[mec-3p::cmk-1(K71A/R74Q/R77S/V292A/V294A)::mNG::3xFlag::unc-54 3’UTR]* was created through a LR recombination reaction between dg68, dg658, dg397 and pDEST-R4-P3.  **dg609***[mec-3p::cmk-1(T179D)::mNG::3xFlag::unc-54 3’UTR]* was created through a LR recombination reaction between dg68, dg592, dg397 and pDEST-R4-P3.  **dg614***[mec-3p::cmk-1(T179A)::mNG::3xFlag::unc-54 3’UTR]* was created through a LR recombination reaction between dg68, dg214, dg397 and pDEST-R4-P3.  **dg668***[mec-3p::cmk-1(K71A/R74Q/R77S/T179D)::mNG::3xFlag::unc-54 3’UTR]* was created through a LR recombination reaction between dg68, dg661, dg397 and pDEST-R4-P3.  **dg669***[mec-3p::cmk-1(W305S/T179D)::mNG::3xFlag::unc-54 3’UTR]* was created through a LR recombination reaction between dg68, dg662, dg397 and pDEST-R4-P3.  **dg671***[mec-3p::cmk-1(T179A/V292A/V294A)::mNG::3xFlag::unc-54 3’UTR]* was created through a LR recombination reaction between dg68, dg664, dg397 and pDEST-R4-P3.  **dg698***[mec-3p::cmk-1(T179A/W305S)::mNG::3xFlag::unc-54 3’UTR]* was created through a LR recombination reaction between dg68, dg692, dg397 and pDEST-R4-P3.  **dg936***[mec-3p::cmk-1(S325D)::mNG::3xFlag::unc-54 3’UTR]* was created through a LR recombination reaction between dg68, dg933, dg397 and pDEST-R4-P3.  **dg941***[mec-3p::cmk-1(T179D/S325A)::mNG::3xFlag::unc-54 3’UTR]* was created by PCR site-directed mutagenesis from dg609.  **dg1007***[mec-3p::cmk1(T179D /V292A/V294A)::mNG::3xFlag::unc-54 3’UTR]* was created through a LR recombination reaction between dg68, dg998, dg397 and pDEST-R4-P3.  **dg1006***[mec-3p::cmk1(K71A/R74Q/R77S/V292A/V294A/L321A/L323A)::mNG::3xFlag::unc-54 3’UTR]* was created through a LR recombination reaction between dg68, dg1000, dg397 and pDEST-R4-P3.  **dg1005***[mec-3p::cmk1(K71A/R74Q/R77S/V292A/V294A/K307Q)::mNG::3xFlag::unc-54 3’UTR]* was created through a LR recombination reaction between dg68, dg1001, dg397 and pDEST-R4-P3.  **dg305***[QUAS:: ckk-1b:: GFP::unc-54 UTR]* was created through a LR recombination reaction between dg229, dg287, mg208 and pDEST-R4-P3.  **dg369***[QUAS:: ckk-1a:: GFP::unc-54 UTR]* was created through a LR recombination reaction between dg229, dg350, mg208 and pDEST-R4-P3.  **dg1013***[mec-3p::cmk1(K71A/R74Q/R77S/V292A/V294A/S325A)::mNG::3xFlag::unc-54 3’UTR]* was created by PCR site-directed mutagenesis with:  S325A_F: gCcAATAGCAATCGCCTACAGAAACAAGCT  S325_R: GGAGAGACGGAGCATTTGAAGCT  from dg665.  **dg1021***[mec-3p::cmk1(V292A/V294A/S325A)::mNG::3xFlag::unc-54 3’UTR]* was created by PCR site-directed mutagenesis  from dg607. |
